# Foliar Application of *Equisetum arvense* Extract Enhances Growth, Alleviates Lipid Peroxidation and Reduces Proline Accumulation in Tomato Plants Under Salt Stress

**DOI:** 10.3390/plants14030488

**Published:** 2025-02-06

**Authors:** Messaouda Boukhari, Rocío Asencio-Vicedo, Mar Cerdán, Antonio Sánchez-Sánchez, Juana D. Jordá, Borja Ferrández-Gómez

**Affiliations:** 1 Department of Biochemistry and Molecular Biology and Edaphology and Agricultural Chemistry, Faculty of Sciences, University of Alicante, 03080 Alicante, Spain; mb300@alu.ua.es (M.B.); rav26@alu.ua.es (R.A.-V.); mar.cerdan@ua.es (M.C.); antonio.sanchez@ua.es (A.S.-S.); juana.jorda@ua.es (J.D.J.); 2 Multidisciplinary Institute for Environmental Studies “Ramón Margalef”, University of Alicante, 03080 Alicante, Spain

**Keywords:** *Equisetum arvense*, tomato, biostimulant, silicon, phenolic compounds, salt stress

## Abstract

Salinity is a major abiotic stress that affects physiological and biochemical processes in plants, reducing the growth, yield, and quality of crops. This problem has been intensified with the reduction of the cultivated area. This study evaluated the response of hydroponically grown tomato plants under salt stress to foliar applications of *E. arvense* extracts. Macro- and micronutrients, as well as silicon and phenolic compounds, were extracted using magnetic stirring and water reflux methods, the latter being the most effective. To evaluate the efficacy of *E. arvense* extracts, spraying was applied at two different doses: EQ-R-1 (23.6 mg·L^−1^ Si and 0.5 mM phenolic compounds) and EQ-R-2 (5.9 mg·L^−1^ Si and 0.125 mM phenolic compounds). Foliar application of both extracts alleviated salinity effects by reducing sodium uptake. *E. arvense* extracts mitigated oxidative stress by a decrease in electrolyte leakage by 29% and malondialdehyde and H_2_O_2_ concentrations by 69% and 39%, respectively, for the extract with the lowest dose. In addition, EQ-R-2 was also more effective by reducing 51.5% proline accumulation. These findings showed the potential use of *E. arvense* extracts as biostimulants to enhance plant tolerance to salinity providing new perspectives in agricultural systems.

## 1. Introduction

The horsetail plant (*Equisetum arvense* L.) is a species of the genus Equisetum belonging to the Equisetaceae family. It includes about 30 species and is characterized as a perennial plant with rhizomes, which have gametophytic and sporophytic life stages. *E. arvense* plant is found in Europe, Asia, North Africa, South America, the USA, and Canada [1,2,3,4].

This group of plants has already been used in traditional medicine for a long time and is recognized in the European pharmacopeia [5,6,7]. Phytochemical studies carried out on *E. arvense* have identified several nutrients and beneficial elements including Na, K, Ca, Mg, P, Fe, Zn, Cu, Mn, Si, Sr, and Ti. Moreover, *E. arvense* has a great variety of bioactive compounds and secondary metabolites including phenolic acids, coumarins, phytosterols, stilbenes, condensed tannins, lignin, saponins, alkaloids, glycosides, carotenes, vitamins, and organic acids [3,6,7,8,9,10]. These molecules are important since they have antifungal, antimicrobial, antibacterial, antiparasitic, antioxidant, nervous and cardiac system protective, dermatological, diuretic, immunological, and anti-inflammatory properties that can be useful against health problems such as chronic musculoskeletal pain, anemia, brittle nail syndrome, periodontitis, treatment of urinary infections, healing effects, tuberculosis or liver protection, among others [2,7,9,11,12,13].

Another attribute of horsetail is that it is one of the plants that accumulate the highest amount of silicon (Si) in its shoots [14,15] reaching more than 25% of the dry weight of the plant [16]. Si is the second most abundant element in the Earth’s crust and has great importance in agriculture. It is considered beneficial for plant growth as it strengthens the plant cytoskeleton, optimizes the photosynthesis process, enhances nitrogen fixation, and increases tolerance to abiotic and biotic stress situations by acting as a scavenger of reactive oxygen species (ROS) [3,17,18,19,20]. In addition, Si provides a physical and biochemical defense system that prevents insect attacks [21] and reduces plant sensitivity to drought and salinity [22,23]. It also increases plant biomass production [24] and nutrient uptake [25].

The beginning of the 21^st^ century is marked by global scarcity of water resources and increased salinization of soil and water. In particular, the shortage of good quality water resources in the Mediterranean region is becoming a serious problem for agriculture, due to the risk of salinization of culture media [26]. So, salinity has become one of the most important abiotic stresses, due to its effects on the physiological and biochemical processes of the plant, producing a reduction in growth, yield, and quality of crops [27,28]. The negative effects of salt on plant growth are related to a reduction in the osmotic potential of growing media, specific ion toxicity, and nutrient imbalance [26]. In response to salt stress, plants develop defense mechanisms such as ion homeostasis, osmotic adjustment, and enhancement of antioxidant defense systems. However, prolonged stress may overwhelm these mechanisms [29]. Currently, the use of biostimulants is an effective strategy for mitigating the adverse effects of salinity stress on plant health, as these products enhance plant vitality through various mechanisms, including hormonal stimulation, exopolysaccharide secretion, osmoprotectant accumulation, ion exchange, increasing the activities of antioxidant enzymes [30].

Applications of *E. arvense* have focused on medicine and the pharmaceutical industry, while its use in agriculture is limited and scarcely investigated. From published studies, it is worth highlighting those conducted by Garcia et al., [31] in which the application of *E. arvense* extracts produced an inhibition of mycotoxin and mildew development in plants subjected to excess water, and a resistance of plants attacked by different types of pathogens or herbivorous insects, respectively. It has also been shown that foliar spraying with a horsetail extract increased the yield and production of basil and its essential oils [16]. In addition, another application studied is the use of *E. arvense* weed as a Si fertilizer in the cultivation of wheat plants [17].

Based on the fact that applications of *E. arvense* extracts have not been addressed to reduce the effects caused by biotic and abiotic stress in horticultural crops, this work proposes the application of horsetail extracts as a biostimulant due to the high concentration of Si and bioactive compounds to reduce the effects caused by salinity on tomato plant growth. In particular, the objectives of this study were: (1) to obtain and characterize botanical extracts of *E. arvense* with a high concentration of phenolic compounds and Si through the optimization of the extraction process, (2) to determine the damage that salinity stress produces in tomato plants, (3) to evaluate the efficacy of foliar application of an extract of *E. arvense* on tomato plants grown under salt stress conditions in a hydroponic system with nutrient solutions that simulate the characteristics of the waters we find in arid environments, and (4) to determine the most appropriate dose of *E. arvense* extracts to alleviate physiological damage in plants subjected to salt stress.

## 2. Results

### 2.1. Optimization of Equisetum arvense Extraction Method

The analysis of macro- and micronutrients, as well as the concentration of Si and total phenolic compounds of aqueous horsetail extracts obtained from the two extraction methods are reported in Table 1. The extraction by water reflux (EQ-R) was able to solubilize the highest amount of nutrients compared to the magnetic stirrer (EQ-M) method, especially for K, Fe, Cu, and Zn. Furthermore, EQ-R extract had a high yield for Si and phenolic compounds compared to EQ-M. It increased Si by 180% and phenolic compounds by 81% with respect to EQ-M extraction.

As for the comparison between EQ-M and EQ-R, it is important to highlight the differences in total phenolic compounds and Si content because both play an important key role in enhancing nutrition efficiency and abiotic stress in plants [32,33,34]. For these reasons, in this work, the main factor to choose the most adequate extraction method was the concentration of Si and phenolic compounds, since there is strong evidence that these active substances can act as biostimulants in plants developed under salt stress conditions and alleviate its effects. According to the results shown in Table 1, the EQ-R extract presented the best nutritional parameters and phenolic compounds and, therefore, was the one applied to tomato plants under salt stress.

### 2.2. Effect of Equisetum arvense Extract on Tomato Plants Growth, Mineral Analysis, Photosynthetic Pigments and Residual Transpiration Rates

Table 2 shows the results obtained for the parameters of plant growth, concentration of macronutrients, micronutrients, and Si, as well as photosynthetic pigments and transpiration intensity in tomato plants subjected to the treatments applied. Aerial fresh weight (AFW), for EQ-R-1 and EQ-R-2 treatments showed values 1.3 and 1.4 times higher, respectively, compared to salt control, and only slightly lower than that of the non-saline control (Table 2). In contrast, no significant differences were noted in root fresh weight (RFW) between plants treated with botanical extracts at the evaluated doses, although an improvement in root growth was observed relative to salt control. Moreover, the Saline control plants showed lower ADW and RDW, with a decrease of 43% and 51%, respectively, compared to the non-saline control. It should be noted that the treatment with *E. arvense* extract significantly improved the ADW, although it did not reach the value of the non-saline control treatment. However, the application of horsetail extracts did not have the same effect on RDW as observed for AFW and RFW.

With respect to the macronutrient uptake, a significant reduction in the foliar concentration of Ca, K, and Mg parallel to Na uptake was found (Table 2). According to the results reported (Table 2), the salt control plants had a foliar Na concentration 5.4 times higher than the non-saline control plants. In contrast to Na, K concentration was reduced from a concentration of 3.82% K for the control plants to 2.36% K for the saline control plants (Table 2).

On the other hand, in the case of Ca^2+^ and Mg^2+^, their absorption and therefore their concentration were significantly reduced in the salt control plants. Data from Table 2 show that the application of the extracts at both doses significantly reduced the foliar concentrations of Na⁺ and K⁺ in the plants. However, there was no significant effect on the concentrations of Ca²⁺ and Mg²⁺ compared to the salt control. Regarding the foliar concentration of micronutrients in each of the treatments, it was observed that in general their concentration was not significantly affected in salt plants compared to plants grown under normal conditions (Table 2), whereas the diluted extract (EQ-R-2) exhibited a biostimulant effect on the Fe and Mn concentrations. In addition, the high Si content in the plants under salt stress and treated with the *E. arvense* extracts should be highlighted because an increase from 664 to 1276 and 1115 mg·L^−1^ occurred for EQ-R-1 and EQ-R-2, respectively.

Finally, the results obtained for the leaf concentration of chlorophyll and carotenes are shown in Table 2. Comparing the behavior of the non-saline control and salt control, it was observed that the production of both pigments was statistically lower due to salinity stress. The foliar application of horsetail extracts produced an increase in both chlorophyll and carotene content, achieving concentrations statistically equal to the standard control treatment in those plants sprayed with the EQ-R-2 dose.

### 2.3. Effect of Equisetum arvense Extract on Oxidative Stress of Tomato Plants Growth

Many stresses in plants cause an imbalance between the formation and elimination of ROS, causing oxidative damage. Thus, to determine the damage caused due to salt stress, it is necessary to establish the oxidative condition of the plants and membrane integrity by measuring the leakage of electrolyte leakage from plant tissues, as well as the content of H_2_O_2_ and MDA in plant cells.

**Figure 1:**
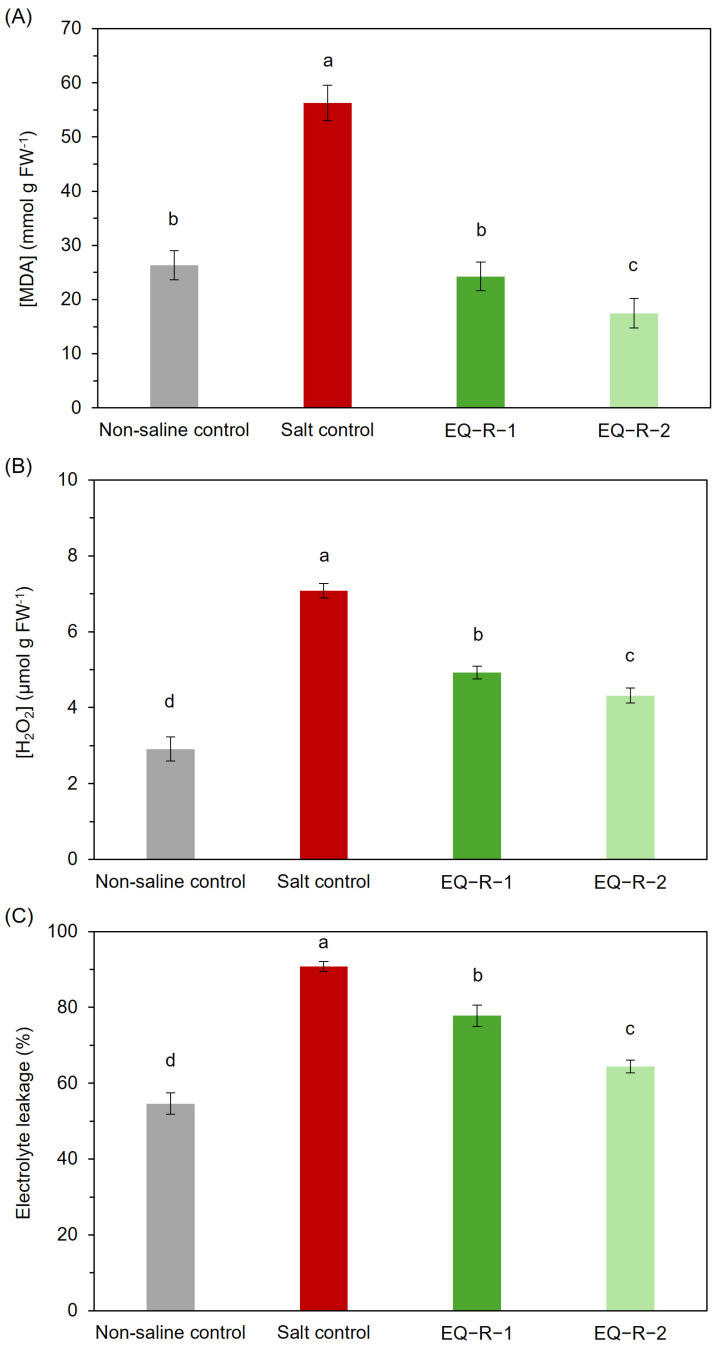
Effect of *E. arvense* extracts with different concentrations on (**A**) MDA content in tomato leaves, (**B**) H_2_O_2_ concentration in tomato leaves, and (**C**) electrolyte leakage in tomato roots grown under salt stress conditions. Means followed by the same letter are not significantly different according to Duncan’s multiple comparison test (*p* < 0.05). The bars show the standard deviation of the mean (n = 5).

Foliar MDA content (Figure 1A) was significantly higher in salt control plants than in the non-saline control plants, 113% higher because of heavy oxidative stress. Foliar application of *E. arvense* extracts to salt-stressed plants reduced the MDA concentration by 57% and 69% for EQ-R-1 and EQ-R-2, respectively, compared to the salt control, and significantly improved the oxidative status of the plants developed, especially the extract of lower concentration (Figure 1A).

Another of the parameters used in this study to evaluate the oxidative stress of the plants was H_2_O_2_ concentration. Saline stress caused an important increase in this molecule in tomato plants (Figure 1B). However, the foliar application of *E. arvense* extract produced a statistically significant reduction in H_2_O_2_. Even though similar to the trend observed with MDA, this decrease was more pronounced at the lower dose (EQ-R-2), with a 39% decrease compared to salt control. The reduction in H_2_O_2_ content indicates that the botanical extract was able to alleviate the damage caused by the oxidative stress caused by the presence of high concentrations of NaCl in the nutrient solution. This behavior correlates with the decrease in MDA content, as a lower H_2_O_2_ concentration in plant tissues results in less oxidative damage and, consequently, less MDA production (Figure 1A).

### 2.4. Effect of Equisetum arvense Extract on Proline Content of Tomato Plants

**Figure 2:**
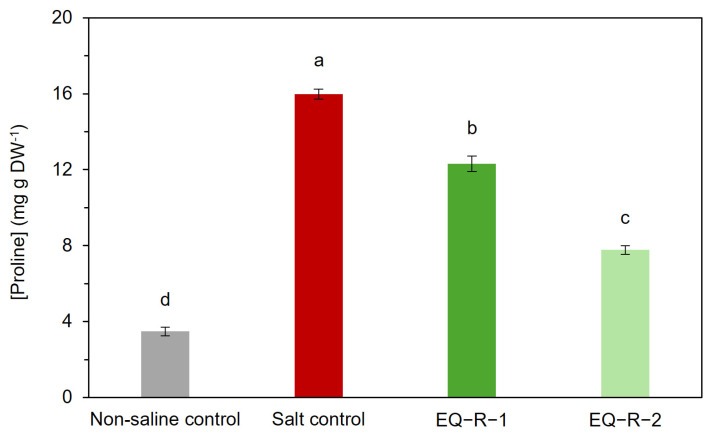
Effect of *E. arvense* extracts with different concentrations on proline concentration in tomato leaves grown under salt stress conditions. Means followed by the same letter are not significantly different according to Duncan’s multiple comparison test (*p* < 0.05). The bars show the standard deviation of the mean (n = 5).

Under salt stress conditions, plants synthesize many low molecular weight organic osmolytes, such as proline. The accumulation of this amino acid is one of the plant’s adaptation mechanisms to salinity and has the advantage of not interfering with normal biochemical or metabolic processes in plant tissues [35]. It is used as an indicator to assess plant tolerance to salinity and to alleviate the inhibitory effects of high ion concentrations on enzymatic activity by stabilizing proteins, protein complexes, or membranes and acting as ROS to reduce oxidative damage in plants under salt stress [35,36]. The results obtained in this study confirm that salt-stressed plants significantly increased proline production, with a concentration four times higher than the non-saline control plants (Figure 2). On the other hand, the application of both doses of horsetail extract (EQ-R-1 and EQ-R-2) resulted in a 22.6% and 51.6% decrease in proline content, respectively, compared to salt control (Figure 2). Accordingly, the foliar application at both doses successfully reduced the proline concentration compared to the salt control, although the content of this osmolyte was not lowered to the levels of plants developed under non-saline conditions.

## 3. Discussion

The results of this work showed that foliar application of *E. arvense* extracts to tomato plants grown under salt stress conditions had a positive effect on plant growth as can be seen in Table 2. It is widely demonstrated that sodium is the main element responsible for the damage caused by salinity and the decrease in K concentration detected in plants is the result of competition with Na [37,38]. These results are in agreement with those obtained by many studies in which it was shown that elevated NaCl levels produce a decrease in AFW and RFW [36,39,40]. This plant growth in saline conditions can be attributed to the effect of Si present in the horsetail extract as previously reported in several studies [22,36,39,40].

In addition, salinity stress resulted in a decline in photosynthetic pigments (total chlorophyll and carotenoids) (Table 2). This was probably due to the structural destruction of the chloroplast and photosynthetic apparatus by the accumulation of reactive oxygen species (ROS) [41]. However, the application of *E. arvense* extract at the highest dose counteracted this effect and even increased the chlorophyll and carotenoid content compared to the salt control. This increase may be due to an enhancement of the synthesis of these pigments as well as a protective effect preventing pigment decomposition. Silicon present in *E. arvense*’s composition would have been a factor in inducing photosynthesis by preventing oxidative damage inside the chloroplasts [42]. Several studies have shown that Si application increases the photosynthetic rate and the pigments by suppressing the level of ROS in plant cells, reducing sodium ion toxicity, and maintaining chloroplast structure and function, which is necessary for the photosynthetic process. Silicon deposition has also been found to provide rigidity to leaves, allowing them to receive more light for photosynthetic activities, further increasing their synthesis [41,43] Nevertheless, the specific mechanisms responsible for resistance to salt stress in chloroplasts by Si supplementation are unknown.

On the other hand, the increase in the photosynthetic pigments content that has been produced in plants treated with *E. arvense* extracts (Table 2) could be also due to the presence of phenolic compounds in the extracts of horsetail, since there are several works that confirm that these compounds have a positive effect on photosynthetic activity, increasing the concentration of plant pigments as well as the improvement of physiological functions associated with the growth of plants [34,44]. This improvement in photosynthetic activity suggested an amelioration of the oxidative stress condition of tomato plants and the integrity of cell membranes, as evidenced by the reduction of both MDA (Figure 1A) and H_2_O_2_ (Figure 1B), resulting in lower lipid peroxidation and reduction of ROS concentration in response to salt stress. It is important to highlight that, in agreement with the decrease in the concentration of these two indicators, it can be seen in Figure 2 that proline also reduces their accumulation. This amino acid is an osmolyte with multiple functions such as maintaining osmotic balance in cells by retaining water and neutralizing ROS.

On the other hand, the use of *E. arvense* extracts could also promote water uptake, thereby diluting the concentration of NaCl in the plant and, in consequence, mitigating its harmful effects. This fact can be observed within the TI results shown in Table 2. Due to the osmotic imbalance in the salt control, there was a closure of the stomata with a consequent decrease in transpiration. These results would be in agreement with those found for other crops grown under salt stress, in which the salinity of the medium also increased stomatal resistance and reduced transpiration [45,46]. The application of botanical extracts (EQ-R-1 and EQ-R-2) had a beneficial effect against salinity stress, as plants treated with these products increased the transpiration rate significantly with respect to the salt control, and in the case of EQ-R-1 treatment, the transpiration rate was restored to the levels of the non-saline control (Table 2). All the treatments with extracts increased TI, probably supporting the lower stress to which they are exposed, with the dilution effect mentioned above.

As for the differences obtained in Na and K concentration, the reduction that can be observed in Table 2 may be due to antagonism between Na^+^ and K^+^ for uptake sites in roots, to the effect of Na^+^ on K^+^ transport in the xylem, or to inhibition of uptake processes [28,47]. As it was reported by Liang et al., [36] the decrease in Ca^2+^ uptake under salt conditions could be due to its precipitation and the increase in the ionic strength that reduces its activity. Moreover, Ca^2+^ availability is reduced by the translocation of Na^+^ to Ca^2+^ at extracellular binding sites [48]. Again, the presence of Si in *E. arvense* extracts could increase K^+^ uptake and transport and decrease Na^+^ uptake and transport from roots to leaves [28,40,47,49], allowing the improvement of all parameters for EQ-R-1 and EQ-R-2 treatments, the less concentrated dose is the one that obtained the best efficiency.

Regarding the results obtained for MDA and H_2_O_2_ concentrations, as mentioned above, their increase observed in stressed plants may be related to the peroxidation of the membrane lipids due to the lower activity of their defense antioxidant enzymes such as catalase, superoxide dismutase, and peroxidase that play a crucial role in ROS scavenging [19,36,50,51]. As a result of these differences, membrane permeability increases. The highest electrolyte leakage values in tomato plant roots were observed again in salt-stressed plants (Figure 1C). Again, the foliar addition of horsetail extracts enhanced the integrity of the cell membranes of the plants grown under salt conditions. A reduction in electrolyte leakage of 15 and 29% was achieved compared to salt control. It is noteworthy that the lower dose extract allowed a higher membrane integrity recovery, although this improvement did not reach the levels observed in the non-saline control plants.

This behavior supports the increase in membrane lipid peroxidation, which, as a result, produces a loss of membrane integrity and, thus, an increase in electrolyte leakage resulting from the saline stress, according to Figure 1A,B, and agrees with the findings reported by several researchers [19,22,36,39,49]. According to these studies, one of the factors contributing to this behavior is Si present in *E. arvense* extracts, as it can increase the antioxidant enzyme activity, thus reducing both the concentration of MDA and H_2_O_2_ in tomato plants under salt stress. In addition to the effect of Si as an abiotic stress alleviator, our results demonstrate a complementary effect with phenolic compounds found in high concentrations in *E. arvense* extracts. This enhanced reduction of oxidative stress can be attributed to the ability of phenolic compounds to induce plant tolerance against stress by improving the activity of enzymatic antioxidants and reducing ROS content [43,52,53]. Consequently, this results in lower MDA and H_2_O_2_ accumulation under saline conditions.

It was demonstrated for both MDA and H_2_O_2_ that the improvement of electrolyte leakage observed in plants treated with *E. arvense* can be attributed to the complementary effect of Si and phenolic compounds. On the one hand, Si application enhances lipid stability within cell membranes, preventing their structural and functional degradation. This is possible because it decreases the peroxidation of membrane lipids by supporting their integrity and reducing membrane permeability [22,45,49,54]. Moreover, the phenolic compounds present in our extracts may have contributed to the reduced membrane permeability by altering the kinetics of peroxidation and improving lipid packing within the membrane [55,56,57]. Consequently, the high composition of Si and phenolic compounds in *E. arvense* extracts contributed to ion uptake and transport, membrane permeability, and, thus, plant growth, mitigating the toxic effects of salt stress.

Finally, these reductions in MDA and H_2_O_2_ concentrations, as well as the lower electrolyte leakage, are directly related to the proline content decrease observed in both EQ-R-1 and EQ-R-2 plants (Figure 1). Proline is an osmolyte that increases in different varieties of plants in response to salt stress. High proline concentrations are indicators of stress as it acts as an osmoregulator, preventing water loss in the plant [58]. In this study, there was no variation in leaf moisture content in any of the treatments, so leaf proline contents were enough to keep the leaf hydrated. Even the highest dose of *E. arvense* extract was the treatment with the highest leaf water content, despite decreasing the proline content. Thus, the decrease in proline seems to be related to a reduction in stress. On the other hand, Munns (2005) described two phases in the salt stress tolerance mechanism [59]. The first is associated with water uptake by the plants which, in the case of tomato, as we have just described, has not been a problem in any of the treatments. The subsequent one is related to the toxic effect of salt and is the one that can damage the cells of the transpiring leaves, reducing their growth. It seems that it is in this second phase that *E. arvense* extracts seem to act, as leaf mass is less reduced, again indicating lower salt stress.

Again, the lower dose (*E. Arvense* extract diluted 1:20 with 5.9 mg·L^−1^ Si and 0.125 mM phenolic compounds) showed a better result. This finding may be due to the high concentration of phenolic compounds applied with EQ-R-1, which may have generated some phytotoxicity, with only the effects of Si being observed, while in the EQ-R-2 treatment, the concentration of phenolic compounds could be more appropriate (Figure 2). According to previous studies, a complementary effect between Si and polyphenols can favor the biostimulant capacity of *E. arvense* [54,60,61].

## 4. Materials and Methods

### 4.1. Plant Material

The vegetative material used in this study was *E. arvense* (horsetail) plant. The dried sample of the *E. arvense* plant was collected from Southeast Spain (September 2023) and purchased (January 2024) in 1 cm length cut stems at a local herb pharmacy of Alicante, Spain (38°25′, 47.428″ N, 0°32′0.491″ W, 189 m above sea level). The voucher specimen was herbarized and deposited in the “Estación Biológica de Torretes—Jardín Botánico de la Universidad de Alicante”, Ibi, Spain (38°63′62.83″ N, 0°53′74.29″ W, 900 m above sea level).

### 4.2. Equisetum arvense Extracts and Characterization

Prior to obtaining the extracts, the botanical sample was ground with an electric grinder until homogeneous size and sieved to a particle size smaller than 2 mm.

Two different methods were carried out to evaluate the effectiveness of the extraction of mineral elements and bioactive compounds. For magnetic stirrer (M) procedure, four grams of *E. arvense* milled leaves were introduced into a spherical flask with 100 mL of distilled water and stirred at 500 rpm for 1 h at room temperature. Afterwards, the extract was filtered through a 0.45 µm filter. This extract was named EQ-M and stored in a dark bottle at 4 °C until use. As for the second method water reflux (R), four grams of *E. arvense* sample were placed in a spherical flask with 100 mL of distilled water and refluxed at boiling point for 1 h. Then, the extract was cooled to room temperature and filtered through a 0.45 µm filter. This extract was named EQ-R and stored in dark bottle at 4 °C until use.

These extracts were analyzed for mineral and total phenolic compounds (Table 1). Macro- and micronutrients, as well as silicon, were performed by inductively coupled plasma mass spectrometry (ICP-MS, 7700×, Agilent, Santa Clara, CA, USA). Phenolic compounds were quantified following the Folin–Ciocalteu method [62]. The extractions using the indicated process, and the characterization were carried out in triplicate.

### 4.3. Cultural Conditions and Treatment

Tomato plants (*Solanum lycopersicum* L. cv. Seny F1, Ramiro Arnedo, Calahorra, Spain) were cultivated in a growth chamber (model MLR-350 H, SANYO, SCLAB, Kyoto, Japan) under controlled conditions of temperature, relative humidity, and light intensity (23 °C, 70%, 250 µE·m^−2^·s^−1^). Seed germination was conducted in silica sand as an inert substrate irrigating them every 2 days with distilled water and a 1 mM CaSO_4_ (Merck KGaA, Darmstadt, Germany) solution to prevent fungal growth. After two weeks, 36 seedlings with a minimum height of 10 cm were transplanted into a hydroponic growing system. Each plant was placed in a 250 mL polyethylene vessel through a hole made on the cover. The composition of the nutrient solution was as follows: 3.5 mM Ca(NO_3_)_2_·4H_2_O, 1.25 mM MgSO_4_·7H_2_O, 4.5 mM KNO_3_, 0.75 mM K_2_SO_4_, 1.5 mM KH_2_PO_4_, 0.5 mM NH_4_NO_3_, 0.31 µM CuSO_4_ 5H_2_O, 1.36 µM ZnSO_4_·7H_2_O, 12.7 µM MnSO_4_·H_2_O, 0.06 µM (NH_4_)_6_Mo_7_O_24_·4H_2_O, 46.3 µM H_3_BO_3_, and Fe which was added in the form of Fe_2_(SO_4_)_3_ (35.8 µM) according to Cerdán et al. [63]. The pH of the nutrient solution was adjusted to 6.0 using KOH (Merck KGaA, Germany) or H_3_PO_4_ (VWR International Eurolab S.L., Barcelona, Spain). In all cases, the roots were immersed in 250 mL of the nutrient solution described above, which was replaced by a new one when the electrical conductivity was ≤0.8 dS·m^−1^.

Plant growth was carried out for 30 days in the greenhouse facilities of the University of Alicante (38°23′05″ N–0°30′47″ W) under controlled conditions of temperature and relative humidity (25/18 °C (day/night) and 70% RH) and 16/8 h light/dark photoperiod. In addition, the greenhouse facilities were equipped with an independent climate control system (air conditioning, heating, a humidifier, and an evaporator) and assimilation lightning. Two days after transplanting, 36 seedlings were randomly distributed into 4 treatments with 9 replicates each. Before the application of the *E. arvense* extract treatments, 27 of the 36 seedlings were exposed to salt stress. For this purpose, the nutrient solution was replaced by a saline nutrient solution, which was prepared by adding 50 mM NaCl (electrical conductivity (EC) = 7.5 dS·m^−1^) to the standard nutrient solution.

According to the results in Table 1, the best extraction process was water reflux during 1 h at 100 °C. However, the high concentration of the phenolic compounds (2.5 mM) and Si (119 mg·L^−1^) in the EQ-R could cause phytotoxicity when applied to young plants [34,64]. To avoid these toxicity problems, two dilution solutions of EQ-R were evaluated to establish their biostimulant effect: EQ-R-1 (1:5 dilution) and EQ-R-2 (1:20 dilution).

To evaluate the effectiveness of the foliar application of EQ-R extract to combat the effects of salt stress in tomato plants, as well as to determine the most effective dose of the botanical extract, the following treatments were established: (i) non-saline control: tomato plants developed under standard conditions and sprayed with 5 mL of distilled water, (ii) salt control: tomato plants developed under salt nutrient solution (50 mM NaCl and EC = 7.5 dS·m^−1^) and sprayed with 5 mL of distilled water, (iii) EQ-R-1: tomato plants developed under salt solution (50 mM NaCl and EC = 7.5 dS·m^−1^ ) and sprayed with 5 mL of *E. Arvense* extract diluted 1:5 (23.6 mg·L^−1^ Si and 0.5 mM phenolic compounds) and, (iv) EQ-R-2: tomato plants grown under salt (50 mM NaCl and EC = 7.5 dS·m^−1^ ) and sprayed with 5 mL of *E. Arvense* extract diluted 1:20 (5.9 mg·L^−1^ Si and 0.125 mM phenolic compounds).

For all plants, the leaves were sprayed, before each foliar treatment, with 5 mL of 1% (*v*/*v*) wetting solution (SpaChem S.L., Valencia, Spain) to improve the penetrability of the active substances. All solutions were applied using hand spray and the treatments were applied once a week for 28 days.

### 4.4. Determination of Plant Growth, Macro- and Micronutrient Concentrations, Photosynthetic Pigment Content and Transpiration Intensity

After 30 days, the tomato plants were harvested and washed with Extran^®^ detergent (Merck, Darmstadt, Germany) to eliminate dust and possible residues of the treatments applied. Subsequently, the plants were washed several times with distilled water. Excess water was removed with lab paper, and plants were weighed to measure their fresh weight for aerial (AFW) and root parts (RFW). After that, samples were dried overnight at 60 °C in an oven and weighed again (ADW and RDW). Finally, the leaf water content (LWC) was calculated [65].

Macro- and micronutrient analyses were performed using 0.3 g of dried leaf samples that were mineralized with HNO_3_ using a microwave wet-oxidized digestion system (Start D, Milestone SRL, Milan, Italy), and the digested samples were diluted in 15 mL of ultrapure water. Nutrient concentration was measured by using an inductively coupled plasma mass emission spectrometry (ICP-MS, model 720-ES, Agilent Technologies, Santa Clara, CA, USA).

Photosynthetic pigments (chlorophylls total and carotenoids) quantification was completed according to the method proposed by Abadía et al. [66]. For this purpose, 1 g of fresh sample was weighed, crushed, and put in a beaker with 25 mL of methanol (Merck KGaA, Germany) and 0.1 g of CaCO_3_ (Merck KGaA, Germany), which was sealed with Parafilm^®^ to avoid evaporation and left to bleach overnight. Afterward, 1 mL of the solution was taken, made up to 25 mL with distilled water, and measured at 665, 652, and 470 nm using a UV–Vis spectrophotometer (JASCO V-630, Jasco Analitica S.L., Madrid, Spain).

Transpiration intensity (TI) was carried out by measuring the water loss of an aerial part of plant of defined surface area over appropriate time intervals [67,68]. For this purpose, one stem was taken from each seedling and was immediately sealed with a small amount of anhydrous lanolin (Cymit Química SL, Barcelona, Spain) and weighed. Afterward, the stems were deposited in an open place and the weight variation was registered every 5 min up to a time of 70 min. Leaf area was determined by drawing the outline of the leaves on pieces of paper, which were weighed and compared with the weight of known areas of the same material. The results were expressed as g·dm^2^·h^−1^.

### 4.5. Malondialdehyde Concentration, H_2_O_2_ Content and Root Electrolyte Leakage

Malondialdehyde (MDA) concentration was determined in foliar samples as an indicator of the oxidative degradation of lipids following the method [69]. A small portion of tomato leaves (0.3 g) were homogenized in 3 mL of trichloroacetic acid 0.1% *v*/*v* (Merck KGaA, Germany). Then, the sample was filtered, and 1 mL was taken and added to a 3 mL mixture in a reaction tube. The solution consisted of trichloroacetic acid 10% *w*/*v* (Merck KGaA, Germany) and thiobarbituric acid 0.65% *w*/*v* (Merck KGaA, Germany) in the same proportion. The reaction tubes were then kept in a thermostatic bath at 95 °C for 25 min. After that, the samples were immersed in an ice bath for 10 min and were centrifuged for 25 min at 12,000 rpm. Supernatant absorbance was measured by UV–Vis spectrophotometry (JASCO V-630, Jasco Analitica S.L., Spain) at 532 and 600 nm.

The method used for the determination of H_2_O_2_ concentration was the colorimetric method with minor modifications [70]. Two hundred micrograms of tomato leaves were homogenized with 3 mL of phosphate buffer (50 mM, pH 6.8, Merck KGaA, Germany). Subsequently, the samples were centrifuged at 5,000 rpm for 25 min. A 3 mL amount of the supernatant was collected and mixed with 1 mL of 0. 1% TiCl_4_ in 20% (*w*/*v*) H_2_SO_4_ (Merck KGaA, Germany) and centrifuged again for 15 min at 5,000 rpm. Quantification was performed by UV–Vis spectrophotometry at 410 nm (JASCO V-630, Jasco Analitica S.L., Spain).

Electrolyte leakage was measured by electrical conductivity. First, the roots were washed with abundant distilled water without separating them from the aerial part and then immersed in a 0.5 mM CaSO_4_ (Merck KGaA, Germany) solution for 30 min to avoid carbonate precipitation on the roots. Subsequently, they were dried with filter paper, and once separated from the aerial part, they were weighed and cut into small segments and placed in centrifuge tubes with 25 mL of distilled water. They were shaken vigorously for 1 min and incubated in a thermostatic bath at 30 °C for 2 h. The electrical conductivity (EC_1_) was then measured, and the samples were re-incubated for 15 min at 100 °C. After this, the tubes were capped with cotton and gauze, then autoclaved at 120 °C for 20 min, and the electrical conductivity (EC_2_) was measured again. The root electrolyte leakage was calculated as the ratio of both electrical conductivities and expressed as a percentage.

### 4.6. Proline Osmolyte Concentration

To measure the proline concentration in the different tomato leaves, the protocol described by Magné and Larher [71] was followed. One hundred micrograms of lyophilized leaves and 10 mL of distilled water at 60 °C were mixed in centrifuge tubes. The sample was homogenized and centrifuged for 15 min at 10,000 rpm at 4 °C. Supernatant was then collected, and 0.5 mL was taken and mixed with 2 mL of acid ninhydrin (Merck KGaA, Germany). They were then incubated again for 30 min at 100 °C and rapidly cooled in an ice bath. Finally, 5 mL of toluene (Merck KGaA, Germany) was added to the samples, which were shaken and kept in darkness until UV–Vis absorbance measurement with a spectrophotometer (JASCO V-630, Jasco Analitica S.L., Spain) at 520 nm.

### 4.7. Statistical Analysis

Results obtained were evaluated using one-factor analysis of variance (ANOVA) with IBM^®^ SPSS^®^ software (23.0 version, IBM, New York, NY, USA). Statistically different groups were determined using Duncan’s test (*p* < 0.05). Prior to the ANOVA analysis, data were tested for normality and homogeneity of variance within Shapiro–Wilk and Levene test, respectively.

## 5. Conclusions

According to our results, the foliar application of *E. arvense* extracts successfully alleviated oxidative and osmotic stress in tomato plants. The increased photosynthetic activity of the plants, as reflected in higher chlorophyll and carotene contents, the highest transpiration rate, and the increase in fresh weight of the treated saline plants compared to the saline plants not treated with the botanical extracts are indicative of the adaptation achieved by the tomato plants to the salinity of the growing medium. At the same time, there is clear evidence that these botanical extracts also reduced the oxidative stress caused by the high NaCl concentration, as the integrity of the root cell plasma membranes was improved, and the proline, MDA, and leaf H_2_O_2_ contents were reduced.

The high concentration of silicon and phenolic compounds in *E arvense* extracts would explain their effectiveness in combating the adverse effects of salt stress. Nevertheless, it should be noted that the lowest dose of this botanical extract was the most effective dose across all parameters due to the lower concentration of compounds phenolic causing possible phytotoxicity.

Overall, the results of the present study suggest that *E. arvense* extracts have the potential as novel biostimulants to mitigate salinity-induced oxidative stress in experiments carried out in hydroponic systems. However, future research is required to determine the optimal concentration of *E. arvense* extracts under different abiotic stress conditions and evaluate their efficacy on various crops. In addition to these variables, further studies on extraction methods of inorganic and organic compounds, including phytochemicals and secondary metabolites, are also needed to optimize and improve the extraction yields.

## Figures and Tables

**Table 1:** Concentration of macro- and micronutrient concentration, silicon, and total phenolic compounds content in *E. arvense* extracts using magnetic stirrer (M) and water reflux (R) method. Results are expressed in average ± standard deviation (n = 3).

Parameter	EQ-M	EQ-R
Nutrient (mg·L^−1^)		
Na	29 ± 3	35 ± 3
K	860 ± 78	931 ± 47
Ca	192 ± 16	170 ± 4
Mg	95 ± 7	91 ± 2
Fe	0.069 ± 0.004	0.15 ± 0.02
Cu	0.062 ± 0.002	0.19 ± 0.03
Zn	0.22 ± 0.03	0.29 ± 0.03
Mn	0.40 ± 0.04	0.36 ± 0.02
Si (mg·L^−1^)	5.9 ± 0.6	118 ± 22
Phenolic compounds (mM)	1.1 ± 0.3	2.6 ± 0.2

**Table 2:** Effect of foliar application of *E. arvense* extracts on plant growth, leaf water content, nutritional and photosynthetic pigments parameters of tomato plants. Data expressed as means ± standard deviation (n = 9) followed by the same letter are not significantly different according to Duncan’s multiple comparison test (*p* < 0.05). Sig. ^1^ is ns: sig > 0.05; *: 0.01 < sig < 0.05; **: 0.001 < sig < 0.01; ***: sig < 0.001.

Parameter	Non-Saline Control	Salt Control	EQ-R-1	EQ-R-2	Sig. ^1^
AFW (g)	23.3 ± 0.3a	12.9 ± 0.3d	16.9 ± 0.4c	21.3 ± 0.4b	***
ADW (g)	2.40 ± 0.05a	1.38 ± 0.17c	1.79 ± 0.14b	2.00 ± 0.16b	***
RFW (g)	7.3 ± 0.2a	3.81 ± 0.09c	3.95 ± 0.11bc	4.3 ± 0.3b	***
RDW (g)	0.51 ± 0.04a	0.25 ± 0.03b	0.28 ± 0.03b	0.32 ± 0.07b	***
LWC (%)	87.0 ± 0.2b	83.5 ± 0.2d	84.4 ± 0.3c	96.5 ± 0.3a	**
Ca (%)	2.69 ± 0.03a	1.82 ± 0.09b	1.68 ± 0.10b	1.76 ± 0.11b	***
K (%)	3.82 ± 0.08a	2.36 ± 0.11c	1.93 ± 0.07d	2.61 ± 0.06b	***
Mg (%)	0.480 ± 0.019a	0.345 ± 0.005b	0.33 ± 0.02b	0.37 ± 0.04b	***
Na (%)	0.33 ± 0.04c	1.79 ± 0.09a	1.53 ± 0.05b	1.48 ± 0.09b	***
Fe (mg·kg^−1^)	122 ± 4a	110 ± 7b	111 ± 5b	120 ± 4ab	*
Cu (mg·kg^−1^)	18 ± 3	16 ± 4	13 ± 2	18 ± 2	ns
Mn (mg·kg^−1^)	116 ± 2a	105 ± 3b	88 ± 4c	102 ± 3b	***
Zn (mg·kg^−1^)	65 ± 3a	68 ± 4ab	55 ± 3c	56 ± 8bc	*
Si (mg·g^−1^)	664 ± 33d	995 ± 29c	1276 ± 58a	1115 ± 55b	***
Chl total (mg·g AFW^−1^)	1.5 ± 0.2a	1.00 ± 0.15b	1.4 ± 0.2ab	1.5 ± 0.3a	*
Carotenoids (mg·gAFW^−1^)	0.35 ± 0.06ab	0.28 ± 0.03b	0.35 ± 0.06ab	0.42 ± 0.4a	*
TI (g·dm^2^·h^−1^)	1.67 ± 0.12a	0.55 ± 0.15c	1.42 ± 0.17a	1.04 ± 0.03b	***

## Data Availability

Data are contained within the article.
